# Effects of hypoxia on anabolic and catabolic gene expression and DNA methylation in OA chondrocytes

**DOI:** 10.1186/1471-2474-15-431

**Published:** 2014-12-15

**Authors:** Karl Alvarez, María C de Andrés, Atsushi Takahashi, Richard O C Oreffo

**Affiliations:** Bone and Joint Research Group, Centre for Human Development, Stem Cells and Regeneration Human Development and Health, Institute of Developmental Sciences, University of Southampton, Southampton, SO16 6YD UK; Department of Orthopaedic Surgery, Tohoku University School of Medicine, Sendai, Japan

**Keywords:** Hypoxia, Cartilage, Epigenetics, DNA methylation, Metabolism

## Abstract

**Background:**

Cartilage is an avascular and aneural tissue. Chondrocytes thrive in this restricted environment of low oxygen tension and poor nutrient availability which has led to suggestions that hypoxia may be a protective mechanism against the development of osteoarthritis (OA). There is also a growing body of evidence to support the role of epigenetic factors in the pathogenesis of OA. However, few studies have investigated the epigenetic-OA process within a hypoxic environment. The current study has investigated the effects of hypoxia on gene expression and DNA methylation of anabolic and catabolic genes involved in the pathogenesis of OA.

**Methods:**

Chondrocytes extracted from OA femoral heads were incubated in normoxia and hypoxia (20% and 2% oxygen concentrations respectively). Interleukin 1-beta (IL-1β) plus oncostatin M (OSM), 5-azadeoxycytidine (5-aza-dC) or media alone (control) were added twice weekly to the incubated samples. After 5 weeks, levels of Collagen type IX (*COL9A1*), *IL1B*, and matrix metalloproteinase-13 (*MMP13*) gene expression were measured using SYBR Green-based qRT-PCR and were correlated with methylation status analysed by pyrosequencing methodology.

**Results:**

Hypoxia resulted in a >50-fold and >10-fold increase in relative expression of *COL9A1* and *IL1B* respectively. This was inversely correlated to the DNA methylation status of these genes. Expression of *MMP13* was reduced at 2% oxygen tension in control cells. Relative expression of *MMP13* increased in cells stimulated with IL-1β and 5-aza-dC in normoxic conditions, and this effect was eliminated at low oxygen tension although no correlation with methylation status was observed.

**Conclusions:**

These findings demonstrate a role for hypoxia in the regulation of anabolic and catabolic gene expression and the influence of changes in DNA methylation. These results further support the role of epigenetics in OA and, critically, highlight the complex relationship between the physiological environment of cartilaginous cells and the osteoarthritic process with implications for therapeutic intervention and our understanding of OA pathophysiology.

**Electronic supplementary material:**

The online version of this article (doi:10.1186/1471-2474-15-431) contains supplementary material, which is available to authorized users.

## Background

Osteoarthritis (OA) is a clinical syndrome characterized by joint pain, functional limitation and often reduced quality of life. Epidemiological studies have shown OA represents one of the leading causes of pain and disability worldwide [1]. An essential feature of the underlying pathogenesis of OA is an imbalance of anabolic and catabolic activity leading to progressive loss and destruction of extracellular matrix (ECM) of articular cartilage [2]. This is exemplified by the aberrant expression of a myriad of catabolic genes, including matrix metalloproteinases (MMPs), aggrecanases (*ADAMTS4* and *ADAMTS5*), inducible nitric oxide synthetase, prostaglandins [3], interleukin-1β (*IL1B*) and a number of cytokines [4], and genes [5, 6].

The influence of genetic susceptibility on the aetiology of OA has been demonstrated in large epidemiological studies, that have characterised OA risk alleles from genome wide association analysis [7]. However, there is also a growing body of evidence to support the role of epigenetics in the pathogenesis of OA. There are three fundamental epigenetic processes: DNA methylation, histone modifications, and non-coding RNAs. DNA methylation is thought to be the principle epigenetic process and has been the subject of intense research [8].

DNA methylation is generally stable in somatic cells throughout adult life [9, 10]. Genes that are expressed in terminally differentiated cells demonstrate hypomethylated DNA corresponding with an open structure allowing the binding of specific transcription factors. In contrast, genes that do not play a role in a cells function are, typically, silenced by hypermethylation [11]. Epigenetic disruption may silence normally expressed genes or induce expression of previously silent genes [12]. This process is thought to be the driver of a number of chronic disease states. Studies have demonstrated hypomethylation at specific CpG sites of catabolic genes in degradative OA chondrocytes [13–15] and, critically, alterations in DNA methylation status in OA may be driven by inflammatory cytokines [11].

Articular cartilage is comprised predominantly of avascular, aneural and alymphatic extracellular matrix, synthesized by sparsely distributed chondrocytes, adapted to oxygen and nutrient limited environments [16]. Low oxygen tensions (<5%) have been shown to promote cell survival when cultured *in vitro* as well as to promote anabolic cell activity and cartilage ECM synthesis compared with culture under normoxic conditions [17, 18]. More recently, modulation of oxygen tensions comparable to that of physiological conditions within human articular cartilage has been shown to reduce the activity of catabolic enzymes implicated in cartilage breakdown such as MMP-1 and MMP-13 [19].

Investigations into the effects of oxygen tension of articular cartilage have to date focused on gene expression of anabolic and catabolic markers, with no reported literature on modulation of the epigenetic processes. The current study has examined the effects of hypoxia on gene expression and DNA methylation, of anabolic and catabolic genes, involved in the pathogenesis of OA.

## Methods

### Cartilage dissection and chondrocyte isolation

Human OA articular cartilage was obtained from 5 patients following total hip arthroplasty (3 men and 2 women with a mean ± SD age of 75.2 ± 7.3 years and an OARSI modified Mankin score of 3–5) [20]. Informed consent was obtained from all patients, and the study was approved by the Southampton & South West Hampshire Local Research Ethics Committee. Only chondrocytes from the superficial layer of OA cartilage were isolated as previously described [21]. Briefly, the cartilage was cut into small fragments and digested by sequential treatment with 10% trypsin (Lonza) in PBS for 30 minutes; 1 mg/ml of hyaluronidase (Sigma-Aldrich) in PBS for 15 minutes and finally collagenase B (Roche Applied Science) in DMEM/F12 (Life Technologies) for 12–15 hours at 37°C.

### Culture and treatment of OA chondrocytes in hypoxia *versus* normoxia

Isolated OA chondrocytes from each donor were divided into three groups and cultured in monolayer: i) control culture, ii) 2 μM 5-azadeoxycytidine (5-aza-dC), and iii) 10 ng/ml of IL-1β plus OSM. Before treatment, chondrocytes were cultured for several days at a density of 2–4 × 10^5^ cells in DMEM/F12 supplemented with 5% of fetal calf serum (FCS; Invitrogen, Paisley, UK), 1% insulin–transferrin–selenium (Sigma-Aldrich), 100 units/ml of penicillin and 100 μg/ml of streptomycin (Lonza), and 100 μg/ml of ascorbic acid (Sigma-Aldrich) in an atmosphere of 5% CO_2_ at a temperature of 37°C. After the initial incubation period and 24 hours in media without FCS to synchronize the cells, each sample was stimulated as per their conditions with DMEM/F12 supplemented with 5% FCS. For the 5-aza-dC group, the histone deacetylase inhibitor trichostatin A (300 n*M*) was added once, at initial treatment, to facilitate access of 5-aza-dC, a cytidine analogue that inhibits the activity of DNMT-1 [22]. This results in the non-specific loss of DNA methylation during cell division. For each condition, one sample was kept at 20% O_2_ tension (atmospheric oxygen tension, normoxia) and one sample cultured at 2% O_2_ tension (hypoxia). The media was changed twice weekly and maintained for 5 weeks until cells reached confluence.

### DNA and RNA extraction

Total RNA and genomic DNA were extracted simultaneously from digested samples using an AllPrep DNA/RNA Mini kit (Qiagen), according to the manufacturer’s instructions. RNA was immediately reverse- transcribed with avian myeloblastosis virus reverse transcriptase and both oligo(dT)_15_ and random primers [23].

### Quantitative reverse transcription –polymerase chain reaction (qRT-PCR)

qRT-PCR was conducted using an ABI Prism 7500 detection system (Applied Biosystems). Exon-exon boundary primers were designed with Primer Express 3.0 software with the exception of *IL1B*, which was designed by and purchased from PrimerDesign Ltd, Southampton, UK. Primer sequences used were *COL9A1* CCTGGTGCTCTTGGTTTGA (F), CACGCTCCCCCTTTTCTC (R); *MMP13* TTAAGGAG- CATGGCGACTTCT (F), CCCAGGAGGAAAAGCATGAG (R); *IL1B* TGGCAATGAGGATGACTTGTTC (F), CTGTAGTGGTGGTCGGAGATT (R) and GAPDH CCAGGTGGTCTCCTCTGACTTC (F), TCATACCAGGAAATGAGCTTGACA (R). The 20 μl reaction mixture was prepared in triplicate, containing 300 ng of complementary DNA, 10 μl of Power SYBR Green PCR Master Mix (Applied Biosystems) and 250 nM of each primer. Thermal cycler conditions included an initial activation step at 95°C for 10 minutes, followed by a 2-step PCR program of 95°C for 15 seconds and 60°C for 60 seconds for 40 cycles. The 2^-ΔΔct^ method was used for relative quantification of gene expression, and the data were normalized to GAPDH expression.

### Analysis of DNA methylation by pyrosequencing

500 ng genomic DNA was bisulfite modified using EZ DNA Methylation-Gold™ Kit (Zymo Research Corporation) according to the manufacturer’s instructions. Promoter regions of interest were amplified using Platinum® PCR Supermix (Invitrogen) and purity confirmed by agarose gel electrophoresis. Percentage DNA methylation was quantified using primers designed with Pyrosequencing™ Assay Design Software Ver 2.0 (Qiagen) and PyroMark™ MD (Qiagen) according to the manufacturer’s instructions. Primer sequences used were *COL9A1* _1 GTTGTTGTGAGAATTAAATGGTATTAAG (F), ACACC- CAACAATCATTATTTATCA (R), CCCAACAATCATTATT- TATC (Seq); *COL9A1* _2 AGGGATTGAAATTTAGGTTGAT (F), AAATTCCAATAAAAATATACCCACTAA (R), GGATT- GAAATTTAGGTTGAT (Seq); *COL9A1* _3 TGAGGGT- TAAAAGTAAAGGGAGAG (F), TTTCCCCTA- TAAATCCCTCCTT (R), GGGAGAGAATTAGAGGTATT (Seq); *MMP13* _1 AATTAGTATTAAGTTTTTTTTTATG- GAAGT (F), TTCAACAAAATCTCAAAACCCATCTAA (R), AAATTTTTTTTTTTTTACCTTCTAT (Seq1), CTCAAAACCCATCTAAC (Seq2); *MMP13* _2 ATGGGTTTT- GAGATTTTG (F), ACCCCTAAATACATCTTAAATA (R), CAATCACTTAAAAATAAACATACTT (Seq1), AATAA- TACCTAAAAACTATTATC (Seq2); *IL1B* _1 ATGGAAGGG- TAAGGAGTAGTAA (F), CCCACATATACTAAATTTAAA- CATTCTT (R), ATACTAAATTTAAACATTCTTCTA (Seq); *IL1B* _2 ATGAAGATTGGTTGAAGAGAATTTTAGA (F), ATTTCTCAACCTCCTACTTCTACTTTTAA (R), ATTTTA- GAGTAGTTTGTTGTG (Seq).

### Statistical analysis

Data is presented as the mean ± SD. Statistical analysis was performed using SPSS software, version 17.0 (SPSS, Chicago, IL) where Wilcoxon’s signed rank test was used to compare between groups. P values less than 0.05 were considered significant.

## Results

### *IL1B* expression is increased in OA chondrocytes cultured under hypoxia and is correlated with loss of methylation

**Figure 1:**
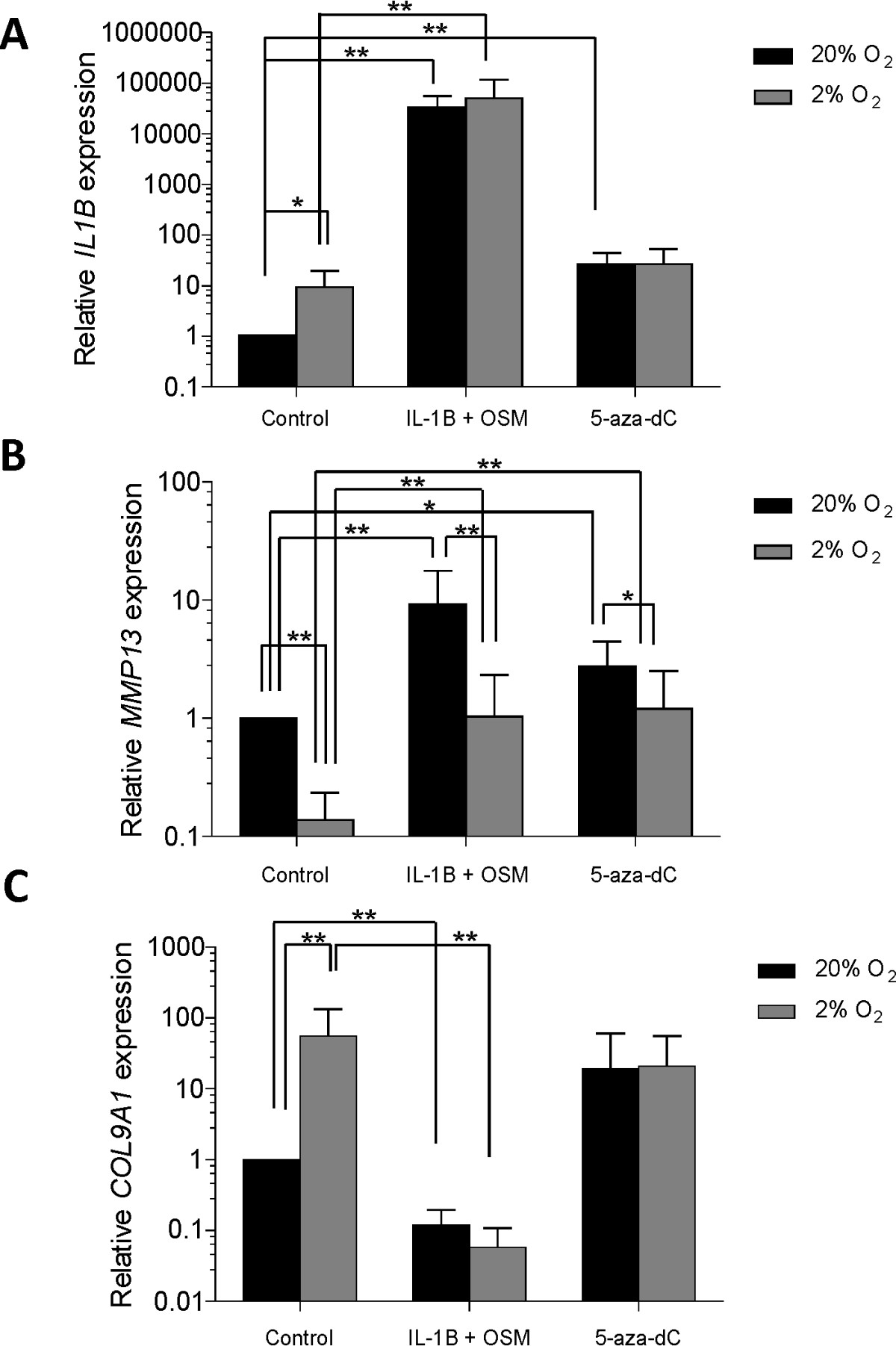
**Relative expression of** ***IL1B*** **(A),** ***MMP13*** **(B) and** ***COL9A1*** **(C) in articular OA chondrocytes under normoxic versus hypoxic conditions analysed by qRT-PCR.** Values are the mean ± SD of triplicate determinations per sample (n = 5; *p < 0.1; **p < 0.05).

As a positive control, OA chondrocytes were cultured for 5 weeks in normoxia and hypoxia, stimulated with the cytokines IL-1β and OSM. As expected there was a significant increase in the relative expression of *IL1B* in these cells compared with controls at both oxygen tensions. This effect was enhanced in hypoxia compared to normoxia (50472 ± 67148 *versus* 32546 ± 23774) although the difference between oxygen tensions did not reach statistical significance (Figure 1A). OA chondrocytes cultured in the absence of IL-1β also demonstrated increased expression of *IL1B* following culture in hypoxia compared with normoxic conditions (9.41 ± 10.23) (Figure 1A).

**Figure 2:**
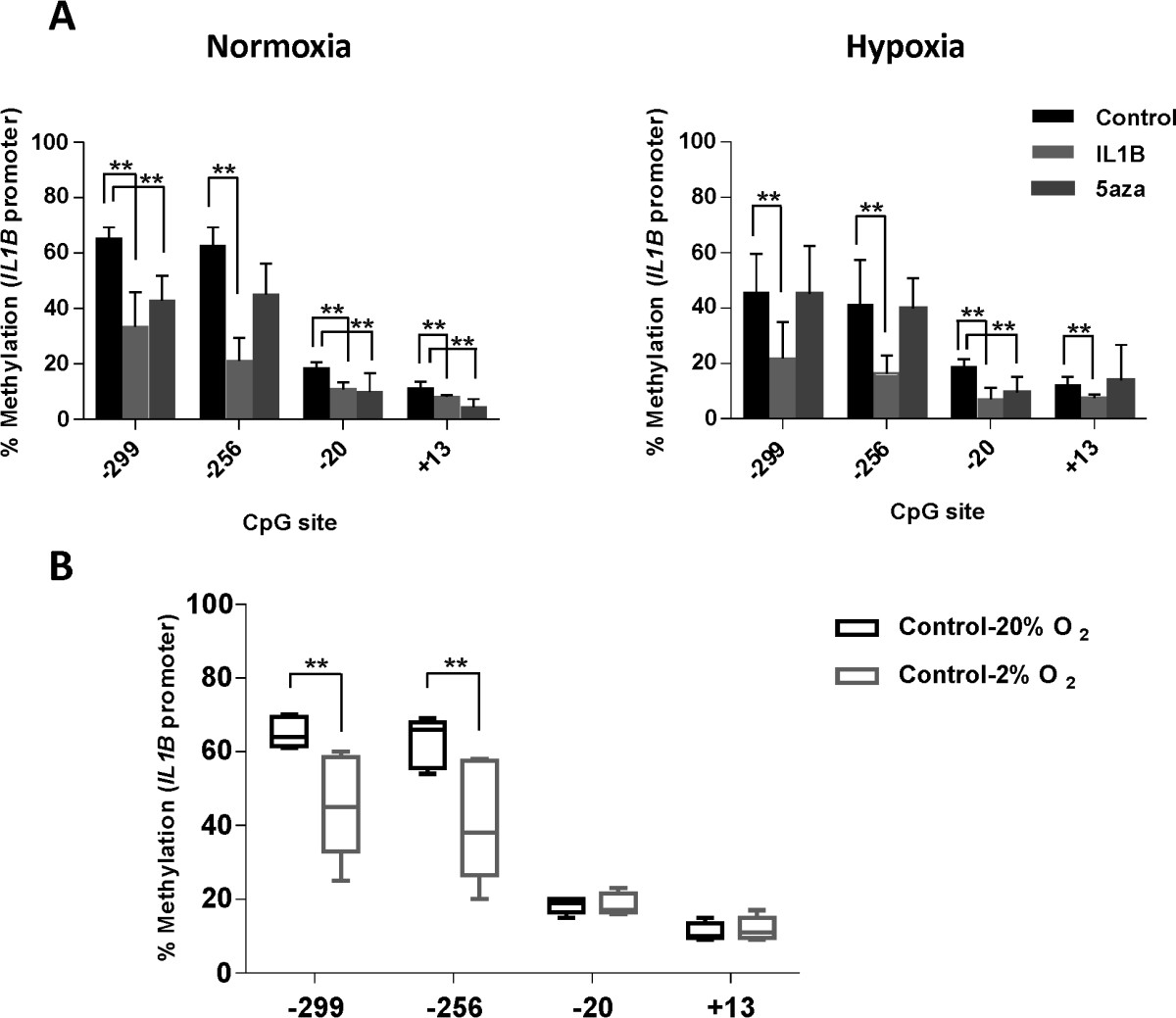
**Methylation status of** ***IL1B*** **proximal promoter in articular OA chondrocytes under normoxic versus hypoxic conditions analysed by pyrosequencing (A); reduction on percentage methylation of specific CpG sites correlates with higher levels of gene expression in OA disease (B).** Primary OA chondrocytes were cultured with or without IL-1β + OSM and 5-aza-dC for 5 weeks. Values are the mean ± SD (n = 5; *p < 0.1; **p < 0.05).

The human *IL1B* promoter contains 7 CpG sites in the 900-bp sequence upstream of exon 1 and 1 CpG site within exon 1 [GenBank: AY137079]. When compared with normoxia, OA chondrocytes cultured in hypoxia displayed a 19.6% reduction and a 21.4% reduction in methylation status at the -299 and -256 CpG sites respectively (Figure 2A-B). This was found to correspond with the changes in gene expression described above. Significant changes in methylation status between oxygen tensions were also observed in cells stimulated with IL-1β at CpG sites -299, -256 and -20 (Figure 2A). Additionally, 5-aza-dc did not alter the methylation status under different oxygen tensions (Figure 2A).

### *MMP-13* expression is decreased in hypoxia conditions in OA chondrocytes and this is not associated with loss of DNA methylation

**Figure 3:**
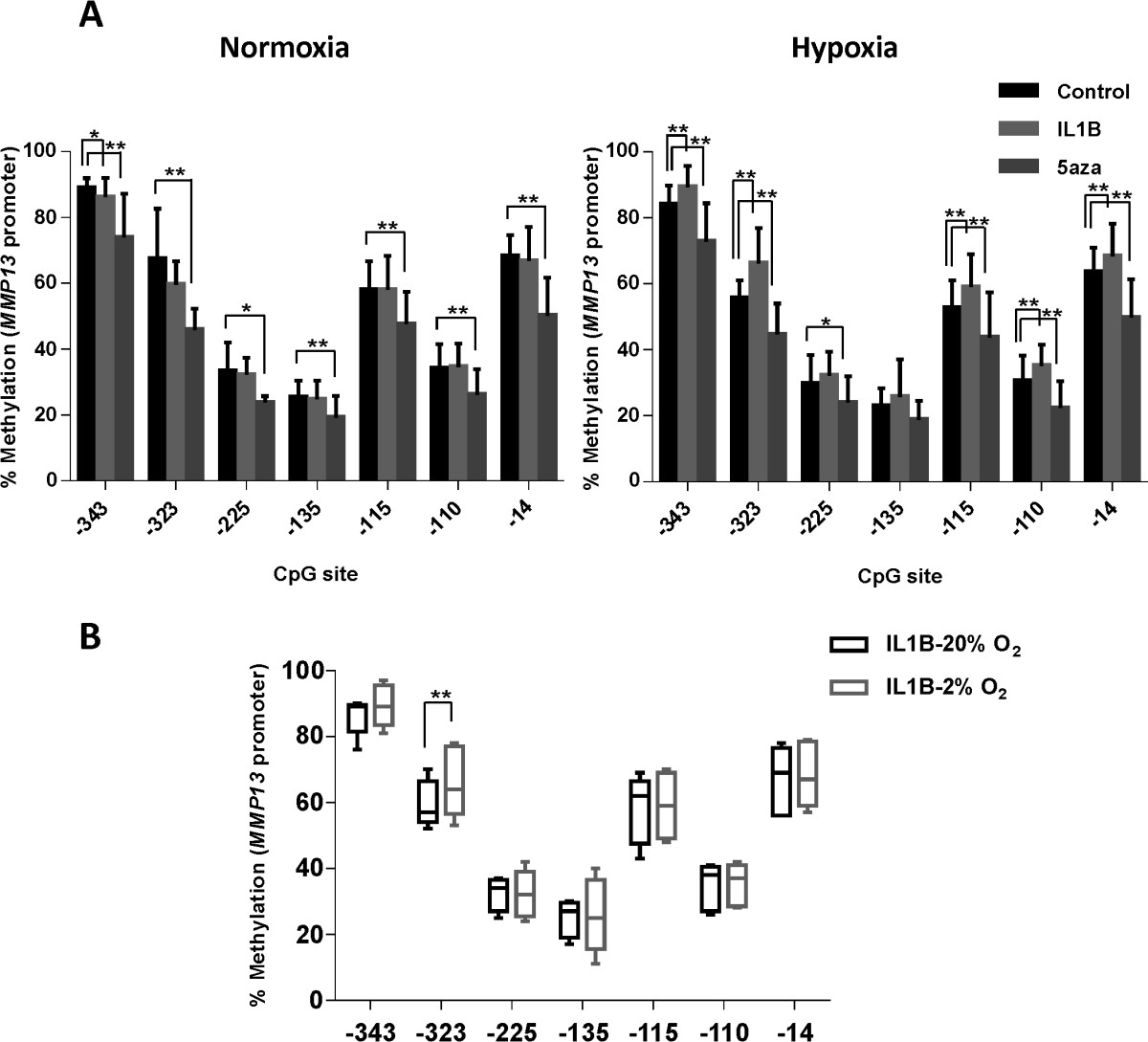
**Methylation status of** ***MMP13*** **proximal promoter in articular OA chondrocytes under normoxic versus hypoxic conditions analysed by pyrosequencing (A); methylation of specific CpG sites correlates with lower levels of gene expression (B).** Primary OA chondrocytes were cultured with or without IL-1β + OSM and 5-aza-dC for 5 weeks. Values are the mean ± SD (n = 5; *p < 0.1; **p < 0.05).

The human *MMP13* promoter has 7 CpG sites in the 400-bp sequence upstream of exon 1 [GenBank: NG021404]; the transcription start site is located 22-bp upstream of the ATG start codon [24]. Relative gene expression of *MMP13* was observed to decrease at physiological oxygen tension compared with normoxia (0.14 ± 0.10) (Figure 1B). This change in gene expression was paralleled by a significant decrease in methylation at 4 CpG sites (Figure 3A).

*MMP13* gene expression was increased in cells stimulated by IL-1β (9.24 ± 8.49) and 5-aza-dC (2.75 ± 1.70) in normoxia. This effect was virtually eliminated in cells cultured in hypoxia (relative expression with IL-1β = 1.03 ± 1.29; relative expression with 5-aza-dc = 1.21 ± 1.30) (Figure 1B). Examination of methylation status of cells stimulated with IL-1β at different oxygen tensions, demonstrated a similar pattern to that of gene expression at all CpG sites except -225 (Figure 3A), however a significant change in methylation status was only observed at CpG site -323 (Figure 3B). Additionally, 5-aza-dc did not alter the methylation status of *MMP13* promoter under different oxygen tensions (Figure 3A).

### *COL9A1* expression is increased in hypoxia with a significant loss of methylation at two specific CpG sites

*COL9A1* relative expression in OA chondrocytes cultured under hypoxic conditions significantly increased in contrast to cells cultured in normoxic conditions (55.92 ± 78.69; Figure 1C). Cells stimulated with 5-aza-dC displayed increased levels of *COL9A1* expression following culture in hypoxic conditions in comparison to culture under normoxia, although these changes did not reach statistical significance. There were no differences in relative gene expression between different oxygen tensions in cells stimulated with IL-1β (Figure 1C).

**Figure 4:**
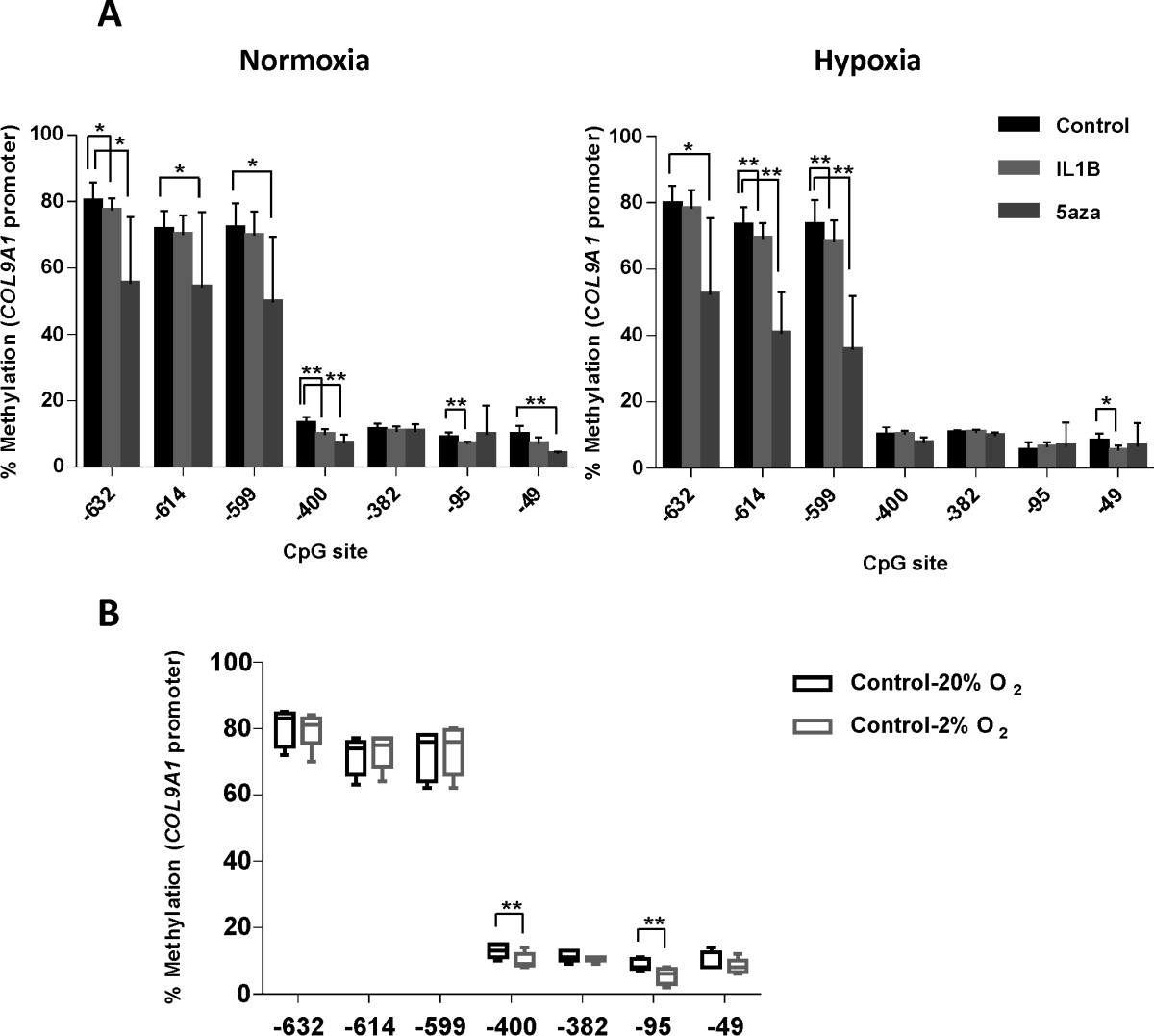
**Methylation status of** ***COL9A1*** **proximal promoter in articular OA chondrocytes under normoxic versus hypoxic conditions analysed by pyrosequencing (A); reduction in percentage of methylation of specific CpG sites correlates with higher levels of gene expression (B).** Primary OA chondrocytes were cultured with or without IL-1β + OSM and 5-aza-dC for 5 weeks. Values are the mean ± SD (n = 5; *p < 0.1; **p < 0.05).

The human *COL9A1* promoter contains 8 CpG sites in the 1,000-bp sequence upstream of exon 1 [GenBank: AF036110] relative to the transcriptional start site [25]. In cells stimulated with 5-aza-dC, a loss of methylation compared with control in normoxia at CpG sites -632, -614, -599, -400, and in hypoxia at CpG sites -632, -614 and -599 was observed (Figure 4A). Comparison of cells stimulated by 5-aza-dC in hypoxia with similar cells in normoxia demonstrated large reductions in methylation at CpG sites -632 (14.0%) and -614 (13.6%) however these changes failed to reach significance (Figure 4A). In addition, there was no correlation between gene expression and methylation status at any CpG sites in control cells or cells stimulated with IL-1β at different oxygen tensions (Figure 4A). Interestingly, a significant loss of methylation was observed at the CpG sites -400 and -95 (13.0 ± 2.1 versus 10.0 ± 2.3 and 8.8 ± 1.6 versus 5.4 ± 2.4 respectively) which may account for the increase in *COL9A1* expression observed in cells cultured under hypoxic conditions (Figure 4B).

## Discussion

The current study has examined the effects of hypoxia on the epigenetic processes governing OA catabolic genes involved in bone modulation and remodelling (*IL1B and MMP13*), and an OA anabolic gene vital in normal cartilage biology (*COL9A1*) [11, 14]. The current study showed that hypoxia resulted in a significant increase in *COL9A1* and *IL1B* expression that was inversely correlated to the DNA methylation status of these genes. In addition, expression of *MMP13* was reduced in 2% oxygen tension in control cells whilst the relative expression of *MMP13* increased in cells stimulated with IL-1β and 5-aza-dC in normoxic conditions, an effect eliminated at low oxygen tension. Previous studies have reported the promotion of anabolic gene expression and the inhibition of catabolic genes in both healthy [19, 26] and OA [27] chondrocytes cultured in hypoxic conditions, leading to the promotion of anabolic and inhibition of destructive processes seen in OA. To date there have been few reports on the relationship between oxygen tension and epigenetic processes in OA. Studer and colleagues in their review, noted that that hypoxic repression of chondrocyte hypertrophy is a combined effect of regulation through different transcription factors as well as the histone deacetylase HDAC4 [28].

Hypoxia inducible factors (HIFs) are transcription factor proteins that have been shown to mediate anabolic and catabolic responses in cartilage. Studies have shown two main isoforms of HIFα promote hypoxia induced cartilage production through direct binding to the master-regulator gene *SOX9* (HIF-2α), and exhibit anti-catabolic responses through down regulation of *MMP13* amongst other collagenases (HIF-1α) [26]. More recently epigenetic processes have been linked with the regulation of HIF-target gene expression in chondrocytes. We previously demonstrated the inhibition of HIF-2α-dependent transactivation in response to the methylation of CpG sites in the hypoxia responsive element in the proximal promoter region of *MMP13*[29].

The anabolic effects of hypoxia on chondrocytes are well documented. Several authors have demonstrated that near cartilage physiological oxygen tensions (<5%) enhanced extracellular matrix synthesis in human articular cartilage [19, 26]. Furthermore, Markway et al. showed no significant difference between healthy and OA cells [27]. The current studies support these earlier findings demonstrating a significant increased expression in *COL9A1* in cells cultured under hypoxic conditions.

A correlation between high levels of *COL9A1* expression and loss of methylation in human fetal bone cells (HFBCs) is known [30] and, interestingly, the degree of demethylation was inversely correlated with developmental age. In the current study limited association between *COL9A1* expression and DNA methylation levels was observed and no correlation between hypoxia and normoxia. These discrepancies may be associated with differences in cells used between the two studies, including the multipotency of HFBCs that is absent in OA chondrocytes.

Less is known about the effect of hypoxia on the regulation of catabolic mechanisms in human articular chondrocytes. Strobel et al. demonstrated an anti-catabolic role of physiological oxygen tension, by the reduction in activity of MMP-1 and MMP-13 [19]. Thoms et al. reported a similar down-regulation of *MMP13* expression by hypoxia; a response mediated directly by HIF-1α. However HIF-1α also resulted in the up regulation of tissue inhibitor of metalloproteinases 3 (TIMP-3), an endogenous inhibitor of MMP-13, representing two mechanisms for the observed changes in gene expression at low oxygen tension [26].

The effects of hypoxia on cytokine expression are less clear. Grimshaw et al. reported a modest increase in *IL1B* and transforming growth factor beta at low (<0.1%) though not higher oxygen tensions [17]. These two factors have antagonistic effects, the former leading to a reduction in aggrecan expression and the stimulation of tissue inhibitors of metalloproteinases (TIMPs) mRNA expression, and the latter a converse effect [31–36]. The current studies support earlier evidence demonstrating a significant reduction in *MMP13* and an increase in *IL1B* expression in OA cells at hypoxia in comparison to normoxia, representing both pro- and anti-catabolic effect. Furthermore, we demonstrate IL-1β induced increased *MMP13* expression under normoxic conditions, suggestive of an overall catabolic effect. We have previously reported evidence that inflammatory cytokines can change the DNA methylation status at key CpG sites leading to the long-term induction of *IL1B* in OA chondrocytes [11, 29]. Here we similarly report a significant decrease in methylation at these key CpG sites (-256 and -299 upstream of the coding start) in cells stimulated by IL-1β compared with controls. Furthermore, a significant decrease in methylation in cells stimulated with IL-1β in hypoxia compared with normoxia was observed, correlating with the increase in expression of *IL1B* seen in cells stimulated with IL-1β and cultured in hypoxia. These results suggest that oxygen tension has a role in epigenetic processes influencing OA cartilage. Whether this is a direct effect or one governed by the effect of the hypoxic environment on other cellular mediators is an area for further investigation. In our previous studies, treatment with the agent 5-aza-dC induced loss of methylation *in vitro* at specific CpG sites accompanied with increased expression of *IL1B*[11], *MMP13*[29] and, *COL9A1*[37] in articular chondrocytes cultured for 5 weeks. However, no significant differences were observed in this regard upon comparison of normoxic versus hypoxic conditions.

The current studies indicate a large degree of inter-donor variation, which may be partly the result of the limited clinical sample size examined. However, similar degrees of variation between samples have been observed in other studies investigating the effect of hypoxia on markers of hypertrophy and degeneration in OA chondrocytes [27]. Further investigation of the effect of hypoxia on gene methylation status would necessitate the use of normal cartilage as well as that of OA donors. This raises further considerations as normal articular cartilage is exposed to a finely regulated gradient of oxygen tension varying from 10% at the surface to around 1% at its deepest layer [38]. This gradient is abolished in OA contributing to the progression of disease [39]. This gradient is important, for example, the observation that mouse cartilage is relatively thin may therefore not provide a sufficient diffusion barrier to create hypoxia [26] is thought to account for interspecies differences observed in studies on the effects of hypoxia on metabolic processes of articular cartilage.

## Conclusions

The current studies indicate hypoxia promotes anabolic processes in OA cartilage and reveal a varied influence on catabolic mechanisms in OA. Investigation into the modulation of articular oxygen tension may aid development of tissue repair and bioengineering strategies in OA including *in vitro* expansion of chondrocytes for cartilage repair, and the targeting of specific molecular modulators of pro-anabolic and anti-catabolic mechanisms in articular cartilage. Critically, these results further support the role of epigenetics as a potential target for the development of strategies for reparation in OA and highlight the complex relationship between the physiological environment of cartilaginous cells and pathophysiology of OA.

## Authors’ original submitted files for images

Below are the links to the authors’ original submitted files for images. Authors’ original file for figure 1 Authors’ original file for figure 2 Authors’ original file for figure 3 Authors’ original file for figure 4

## Abbreviations

5-aza-dC: 5-azadeoxycytidine
COL9A1: Collagen type IX
ECM: Extracellular matrix
FCS: Fetal calf serum
HFBCs: Human fetal bone cells
HIFs: Hypoxia inducible factors
IL-1β: Interleukin 1-beta
MMP-13: Matrix metalloproteinase-13
MMPs: Metalloproteinases
OSM: Oncostatin M
OA: Osteoarthritis
TIMPs: Tissue inhibitors of metalloproteinases
TIMP-3: Tissue inhibitor of metalloproteinases 3
