# Supplementary figures and images for: Effects of hypoxia on anabolic and catabolic gene expression and DNA methylation in OA chondrocytes
Source: BMC Musculoskelet Disord. 2014 Dec 15;15:431. doi: 10.1186/1471-2474-15-431 (PMC4301830; doi:10.1186/1471-2474-15-431)

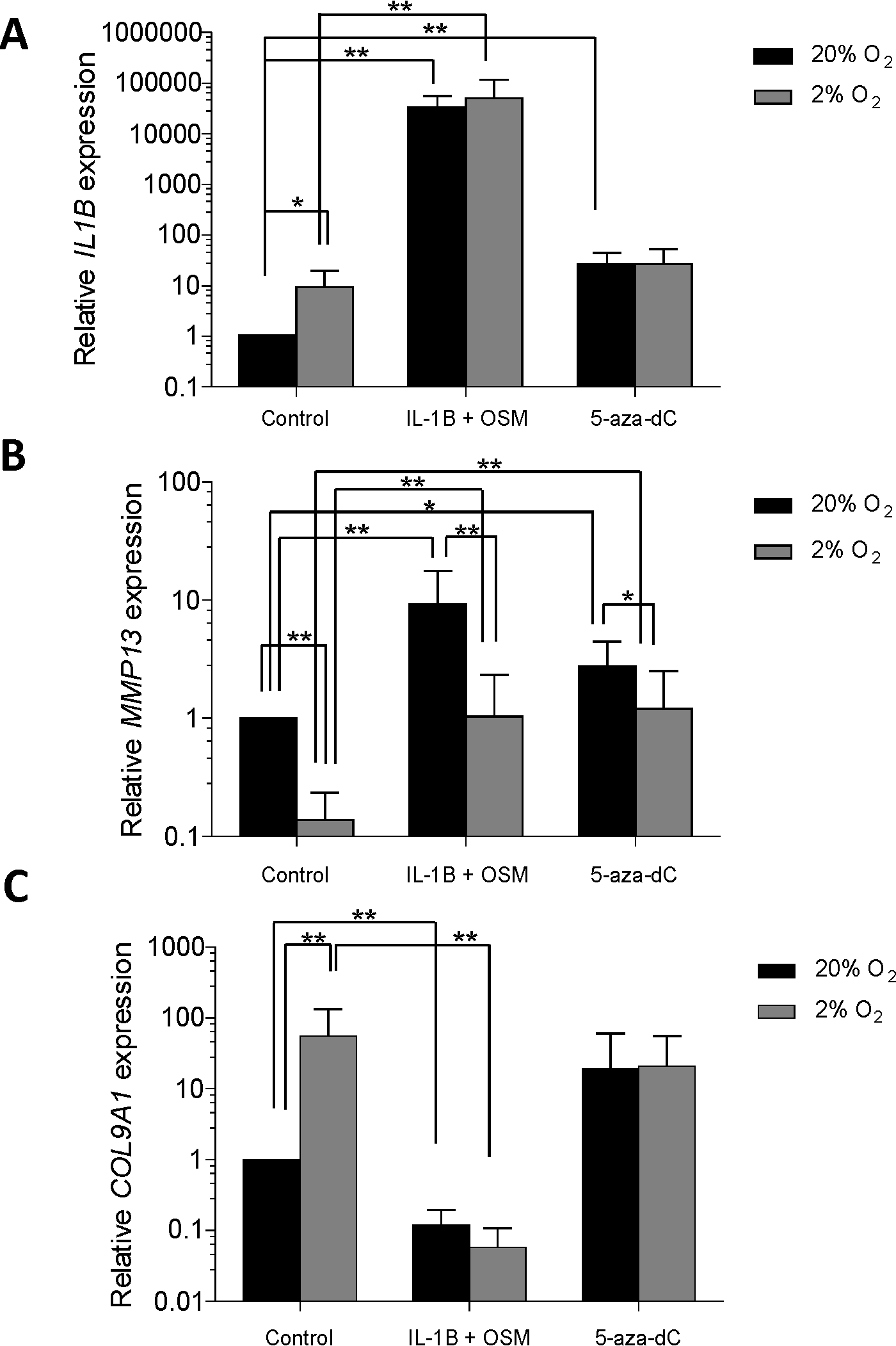

Supplement: Supplementary file 1 — Authors’ original file for figure 1 [file 12891_2014_2377_MOESM1_ESM.tiff]

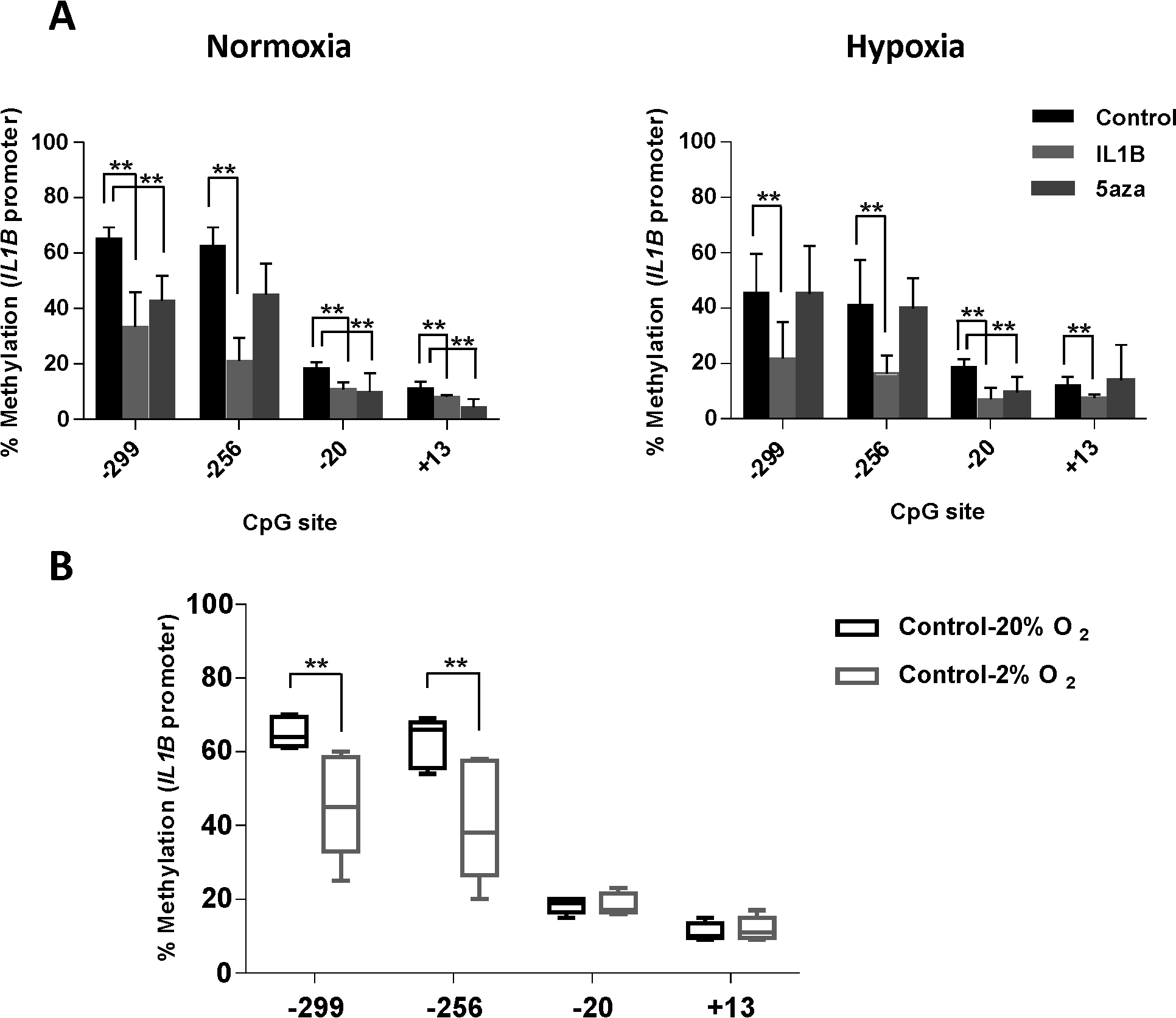

Supplement: Supplementary file 2 — Authors’ original file for figure 2 [file 12891_2014_2377_MOESM2_ESM.tiff]

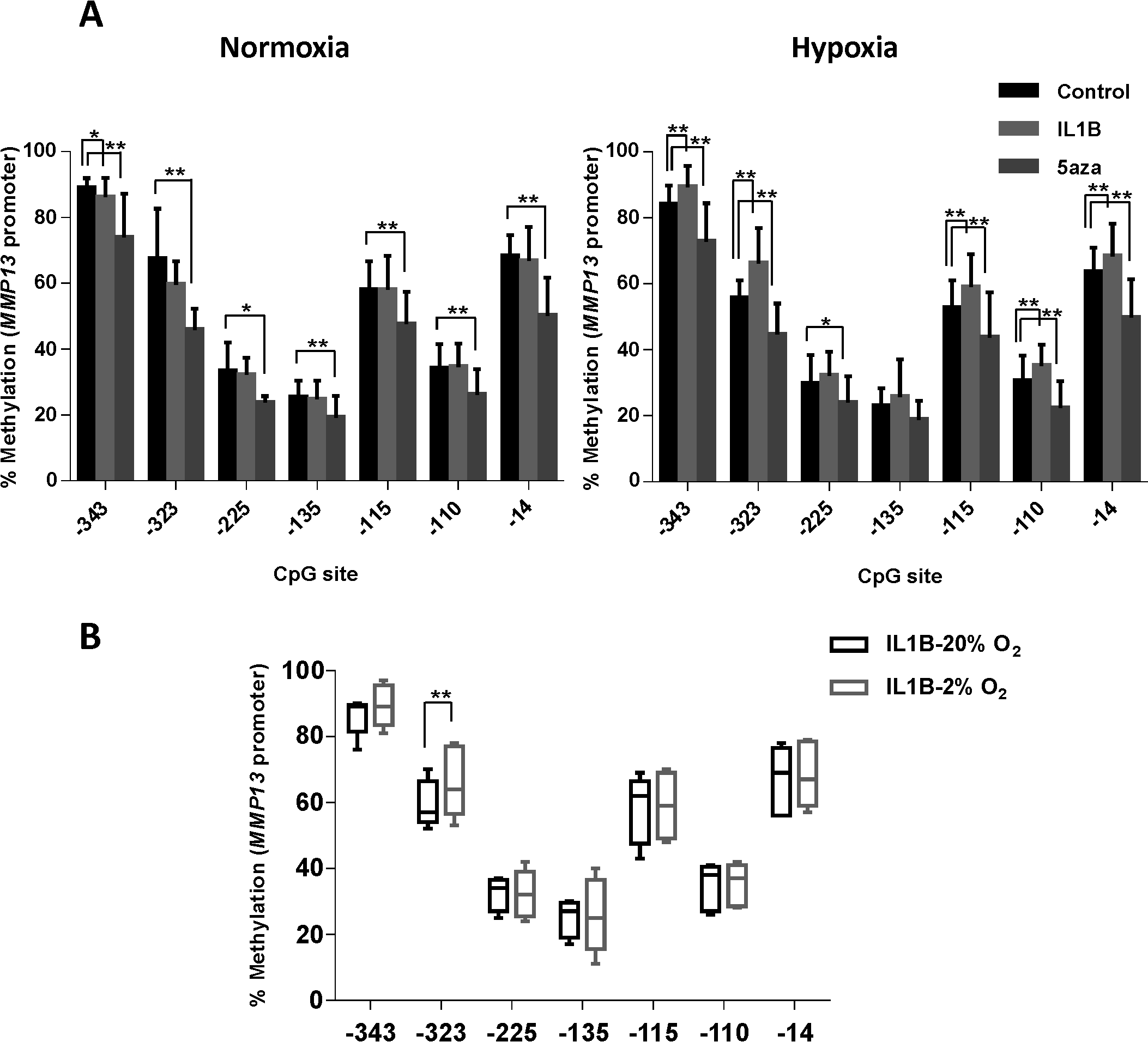

Supplement: Supplementary file 3 — Authors’ original file for figure 3 [file 12891_2014_2377_MOESM3_ESM.tiff]

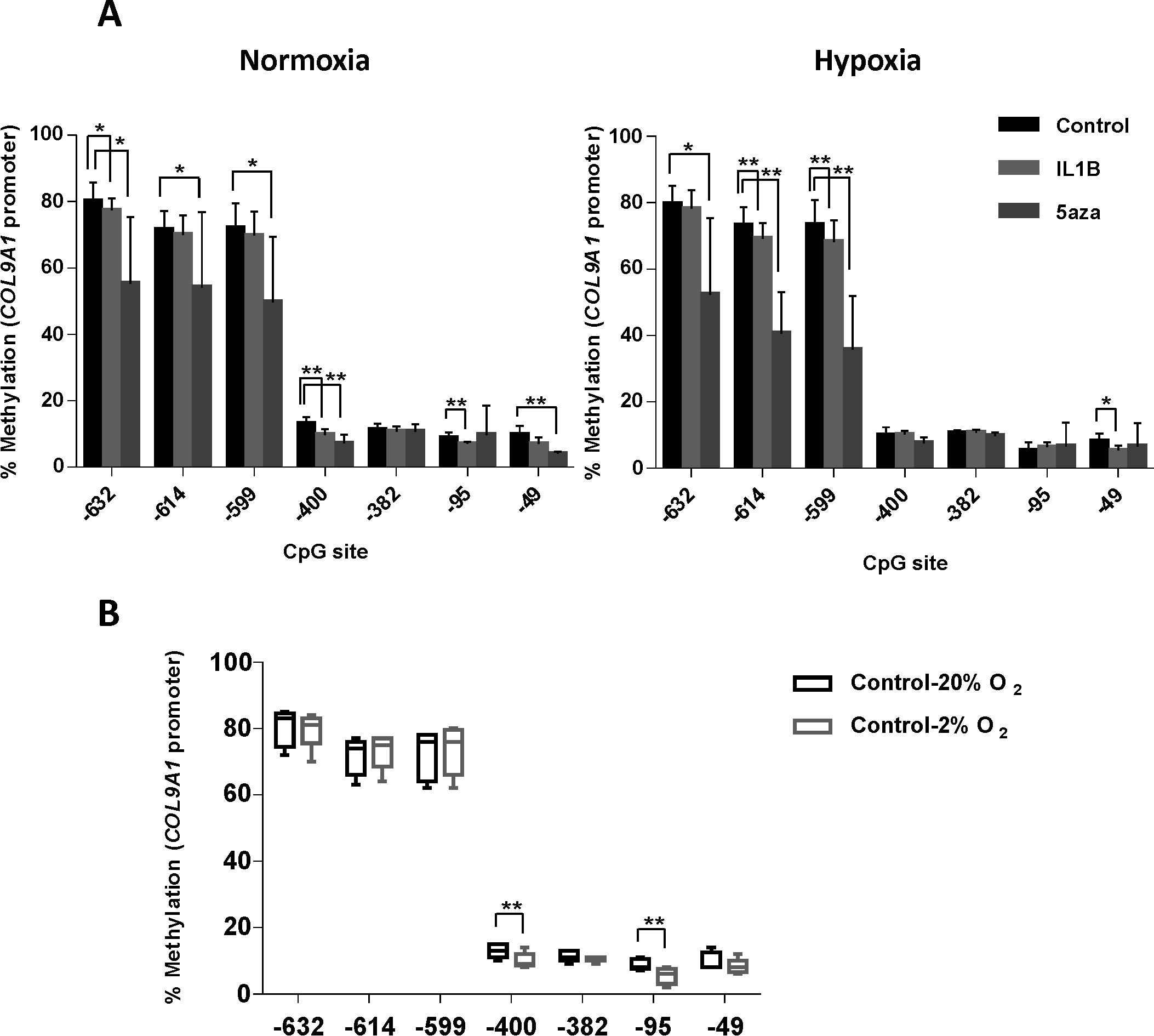

Supplement: Supplementary file 4 — Authors’ original file for figure 4 [file 12891_2014_2377_MOESM4_ESM.tiff]
